# Basal Cell Adenoma of the Lip: A Report of Two Cases and Review of the Literature

**DOI:** 10.7759/cureus.93086

**Published:** 2025-09-24

**Authors:** Loubna Amminou, Maryam Salah, Sara Boukssim, Bassima Chami

**Affiliations:** 1 Oral Surgery, Department of Oral Surgery, Faculty of Dental Medicine in Rabat, Mohammed V University in Rabat, Rabat, MAR

**Keywords:** basal cell adenocarcinoma, basal cell adenoma, immunohistochemistry, oral benign tumor, salivary gland tumor

## Abstract

Basal cell adenoma (BCA) is a tumor usually found in the parotid gland and less frequently in accessory glands. It is seen in the elderly, and it presents in the form of a solitary nodule, well-defined, and covered with healthy mucosa. Histological differential diagnosis should exclude basal cell adenocarcinoma (BCAC), which has histological similarities that may lead to an erroneous diagnosis. We present the cases of two patients diagnosed with BCA who consulted the Oral Surgery Department of the Consultation and Dental Treatment Center of the Ibn Sina University Hospital in Rabat.

## Introduction

Basal cell tumors include basal cell adenoma (BCA), canalicular adenoma, and basal cell adenocarcinoma (BCAC). BCAs are benign tumors characterized by the presence of a palisade of cells with the absence of a myxochondroid stromal component in pleomorphic adenoma. Their occurrence is rare (accounting for 1-3% of all salivary gland tumors), and when present, they essentially affect the major salivary glands, notably the parotid. Their occurrence at the level of the accessory salivary glands is exceedingly rare [1,2]. We report two cases of BCA involving the accessory salivary glands of the upper lip, which represent an unusual site of occurrence.

## Case presentation

Case 1

**Figure 1:**
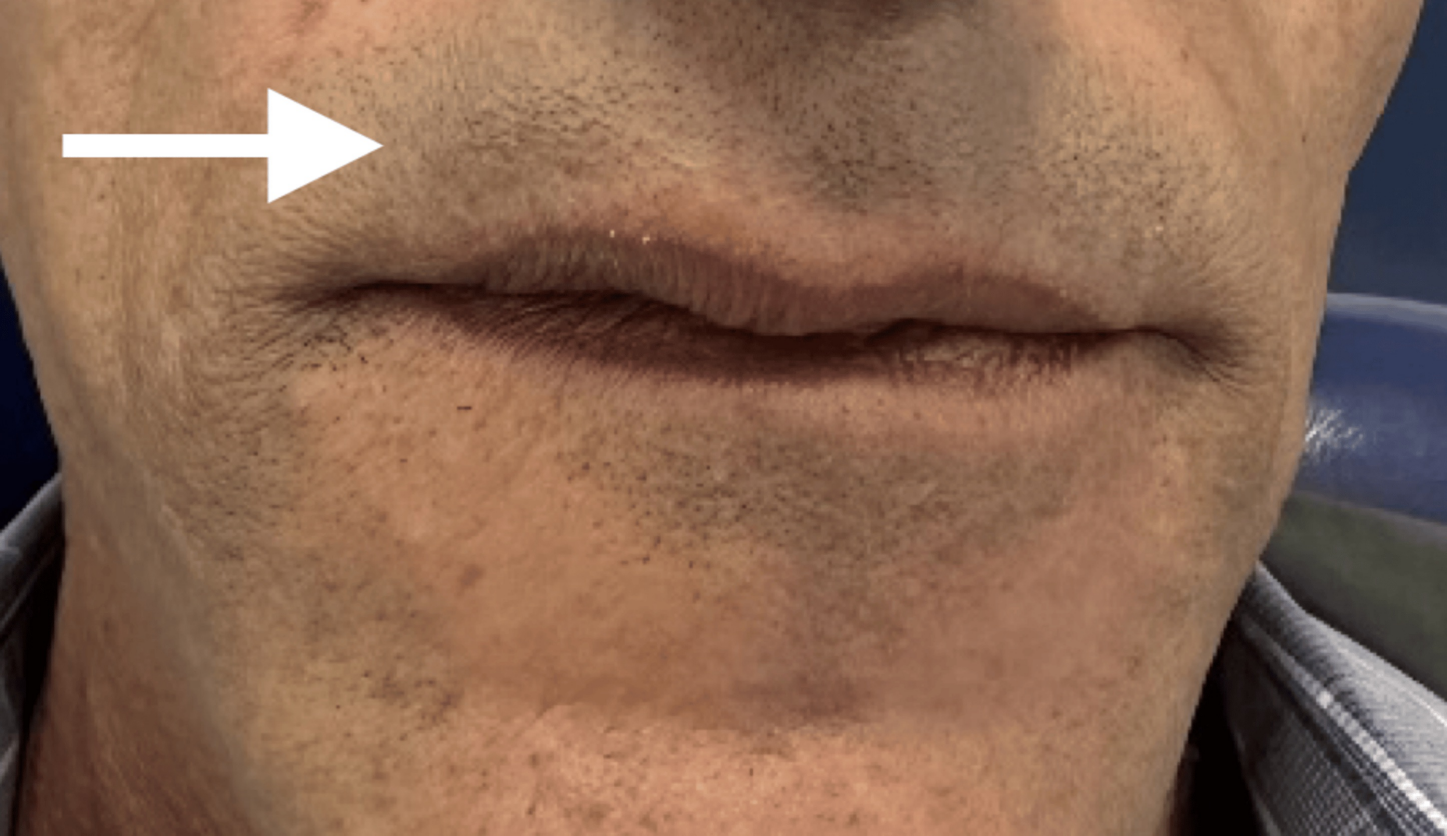
Exobuccal view focusing on the lips The arrow points to swelling that impacts the right side of the upper lip's volume

**Figure 2:**
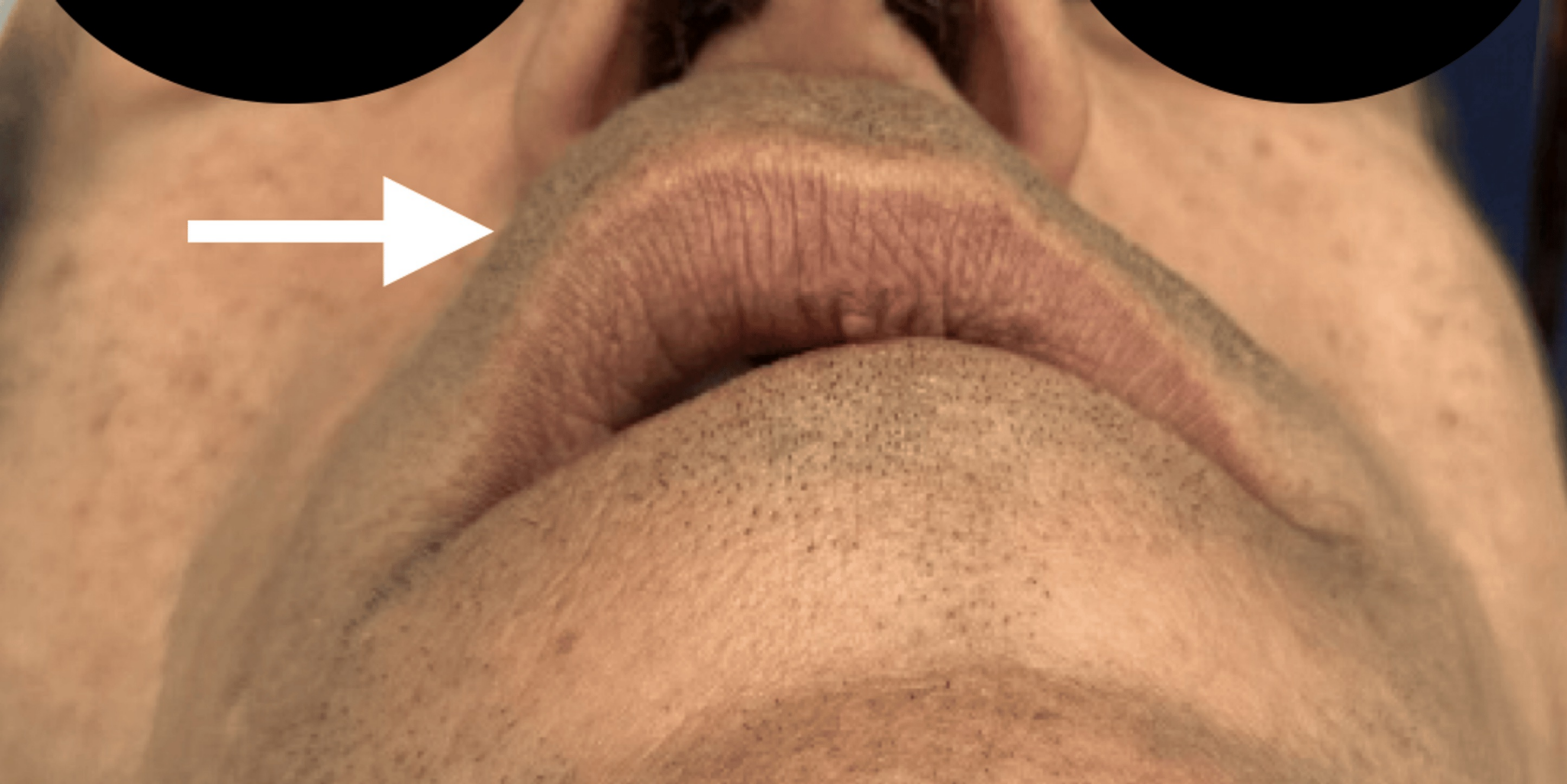
Inferior view of the patient The arrow points to a swelling on the right side, causing an asymmetry

**Figure 3:**
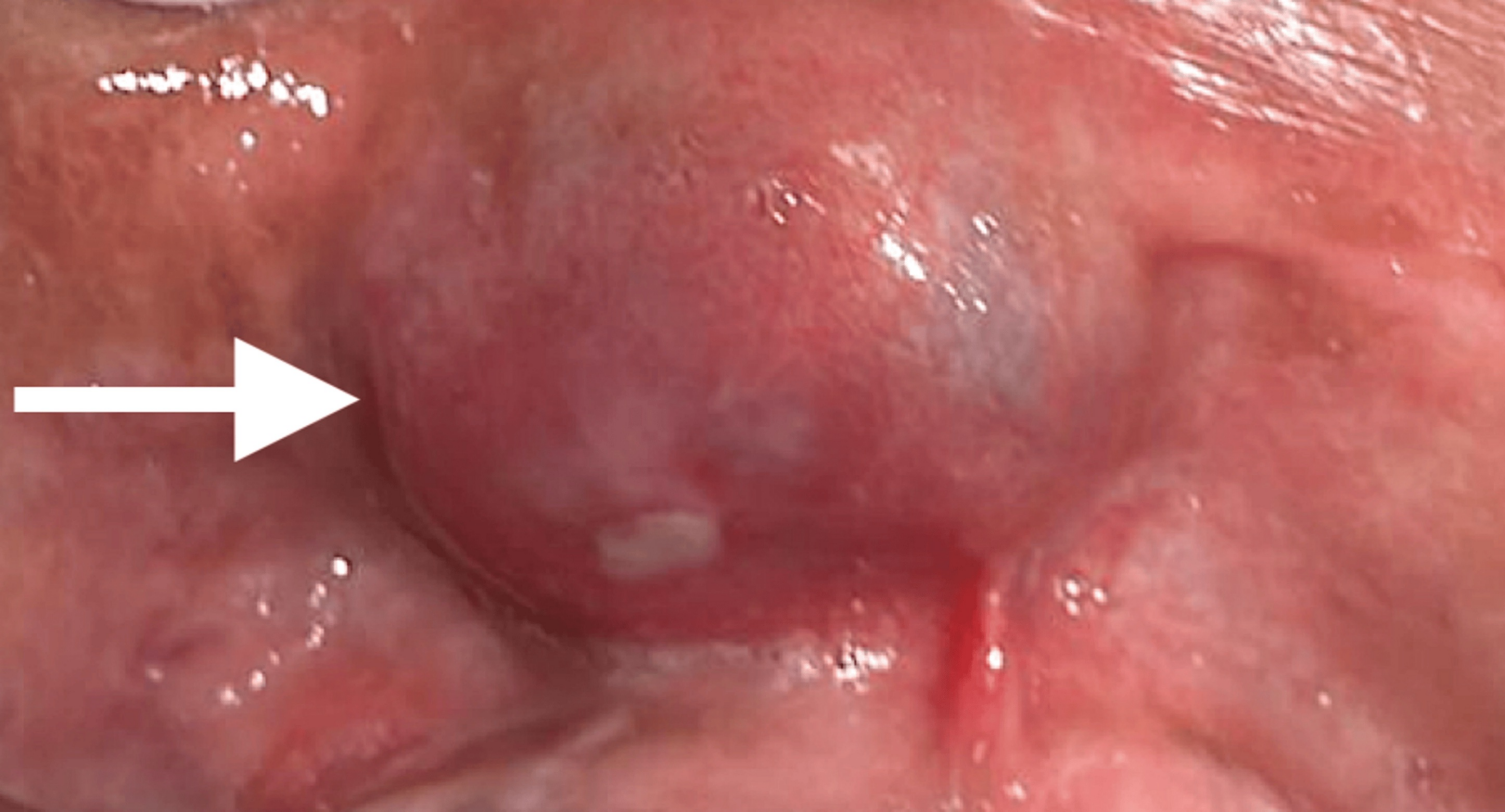
Intraoral view revealing a well-defined swelling in the inner side of the lip The arrow points to a mass covered by normal-appearing mucosa with an area of ulceration

A 58-year-old male with no general health problems consulted for labial swelling on the right side that had been evolving for months, causing noticeable asymmetry and raising concerns for the patient. On exobuccal inspection, we noted a labial asymmetry at the level of the upper lip. The lip covering skin was normal (Figures 1, 2). At the intraoral side, there was a nodule measuring approximately 3 cm, located on the right side of the mucosal surface of the lip, and covered by mucosa with areas of ulceration. The patient wears a total maxillary prosthesis whose edges are in contact with the nodule, which could explain the presence of the ulceration. On palpation, the nodule was painless, firm, well-defined, and slippery under the fingers (Figure 3).

**Figure 4:**
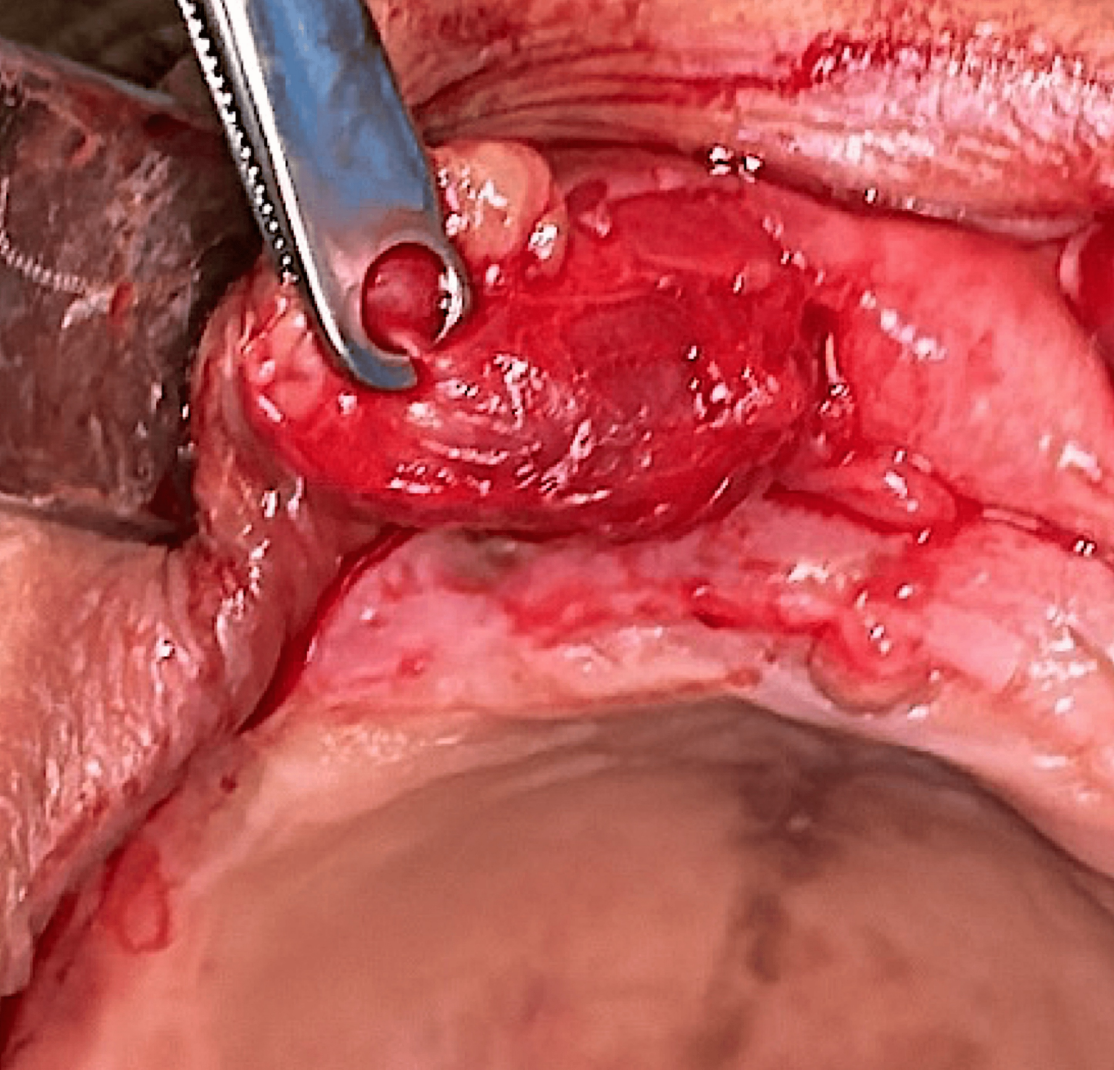
Excision of the lesion

**Figure 5:**
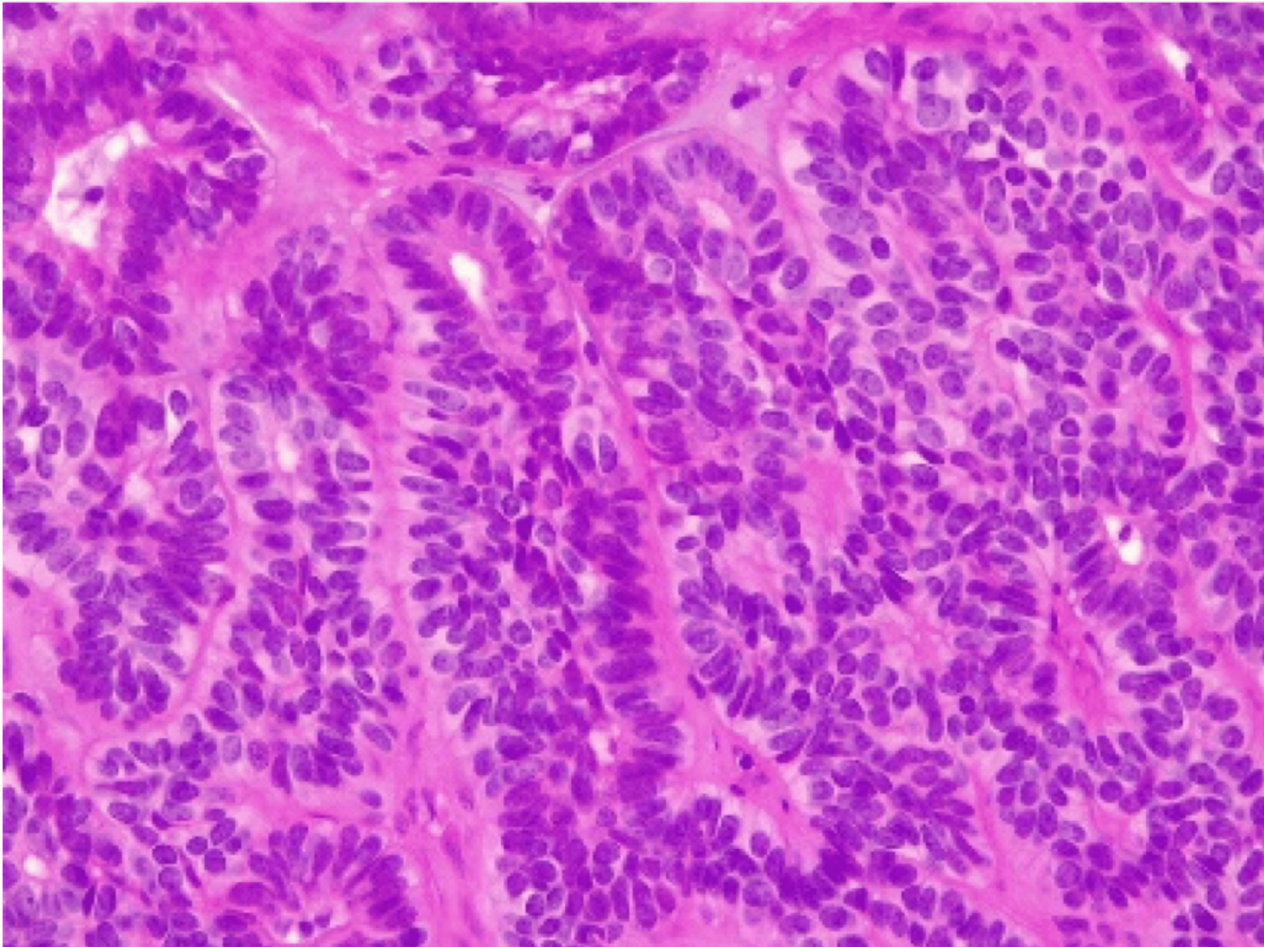
Histological appearance of the lesion (HE X40) showing a well-defined, encapsulated basal cell epithelial proliferation

**Figure 6:**
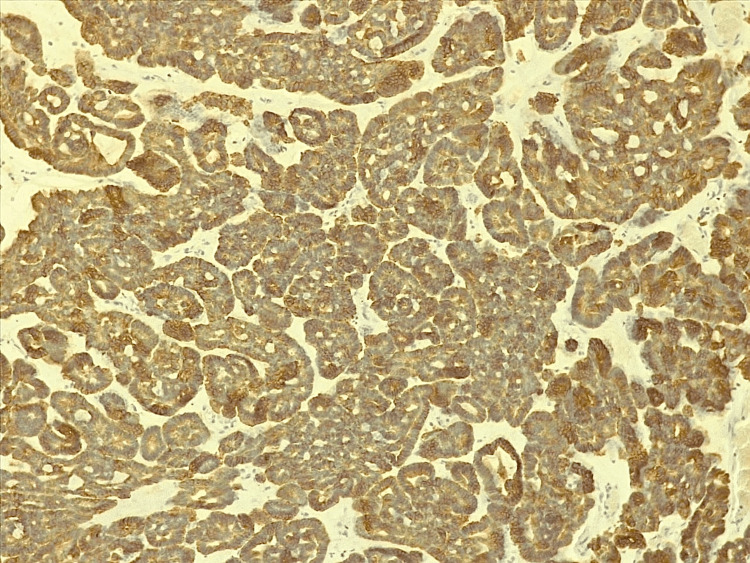
Immunohistochemistry showing the positive dosage of CK7 antibodies

The main histopathological differential diagnoses of BCA include BCAC, adenoid cystic carcinoma, pleomorphic adenoma, and mucoceles. An excisional biopsy under local anesthesia was performed (Figure 4). Histological examination revealed a well-defined, encapsulated basal cell epithelial proliferation (Figure 5). The assessment was further complemented by immunohistochemistry: CK7 staining was positive, highlighting the epithelial nature of the tumor cells, and P63 staining showed strong peripheral positivity, supporting the basal cell phenotype. Immunohistochemical analysis is valuable for differentiation: in our case, the combination of CK7 positivity and peripheral P63 staining confirmed the diagnosis of BCA and helped to rule out malignant tumors and mucoceles, which lack basaloid cell proliferation (Figure 6).

Case 2

**Figure 7:**
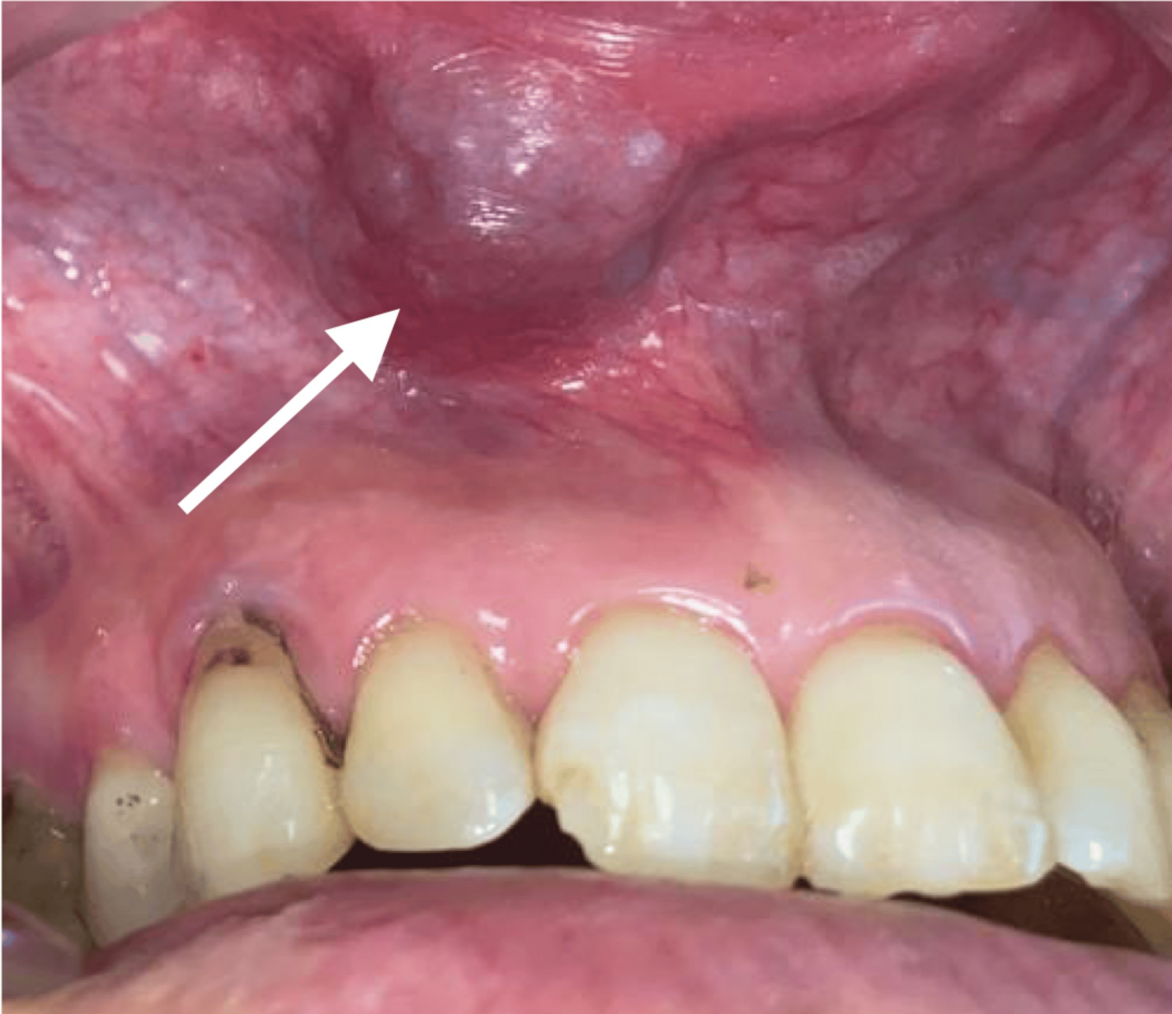
Endobuccal view The arrow points to a swelling in the inner side of the upper lip

A 75-year-old female presented to the oral surgery department with a painless lesion of the upper lip. She reported being in good general health and noted that the lesion had appeared seven months prior. On extraoral examination, the upper lip was noted to be slightly elevated. On inspection, a 2-3 cm nodule was observed in the central portion of the inner aspect of the lip, covered by normal-appearing mucosa. On palpation, the nodule was firm, painless, well-circumscribed, and mobile (Figure 7). No lymphadenopathy or sensory deficits were detected. The clinical differential diagnoses included mucoid cyst, pleomorphic adenoma, and BCA.

**Figure 8:**
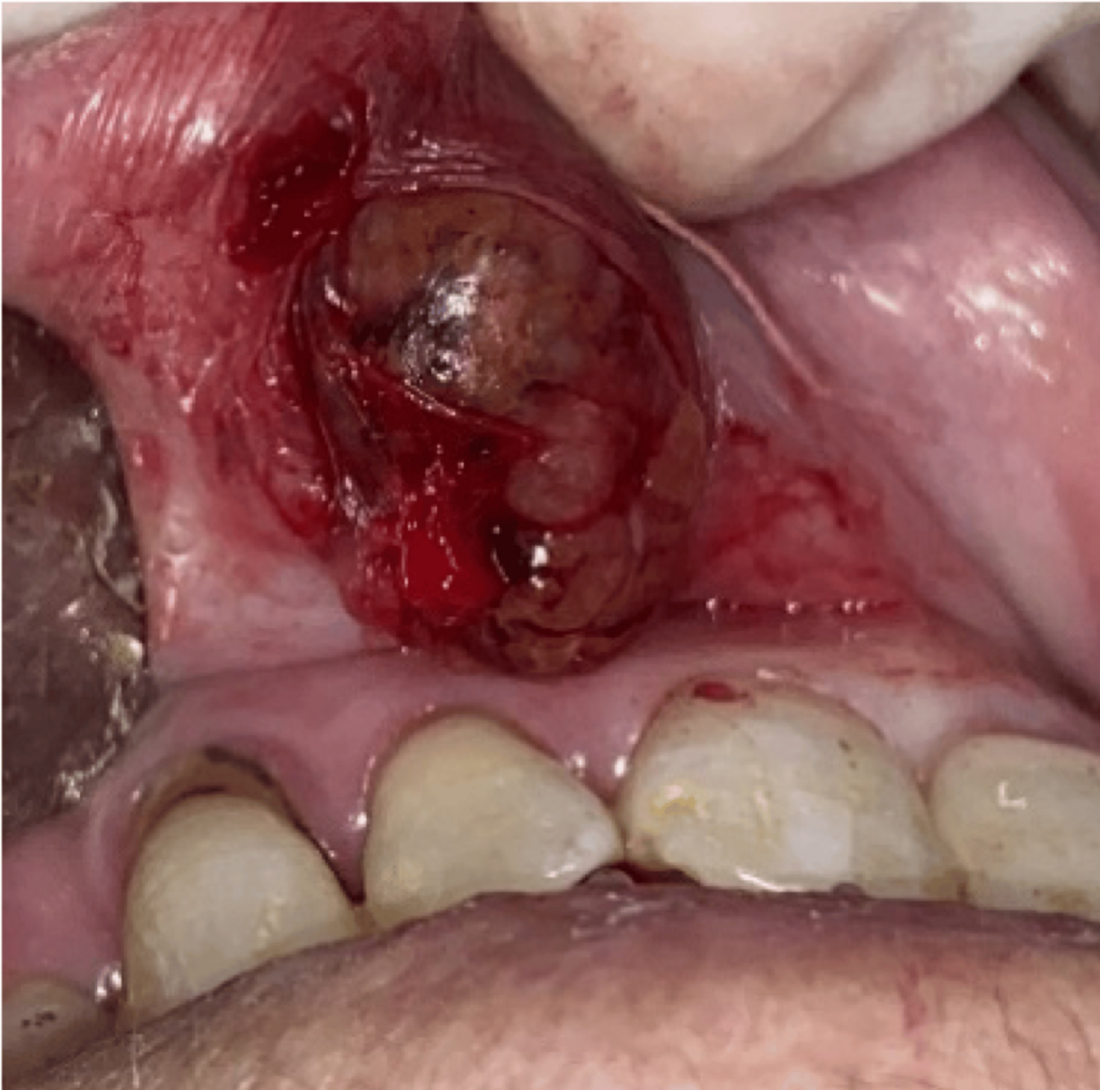
Excision of the lesion

**Figure 9:**
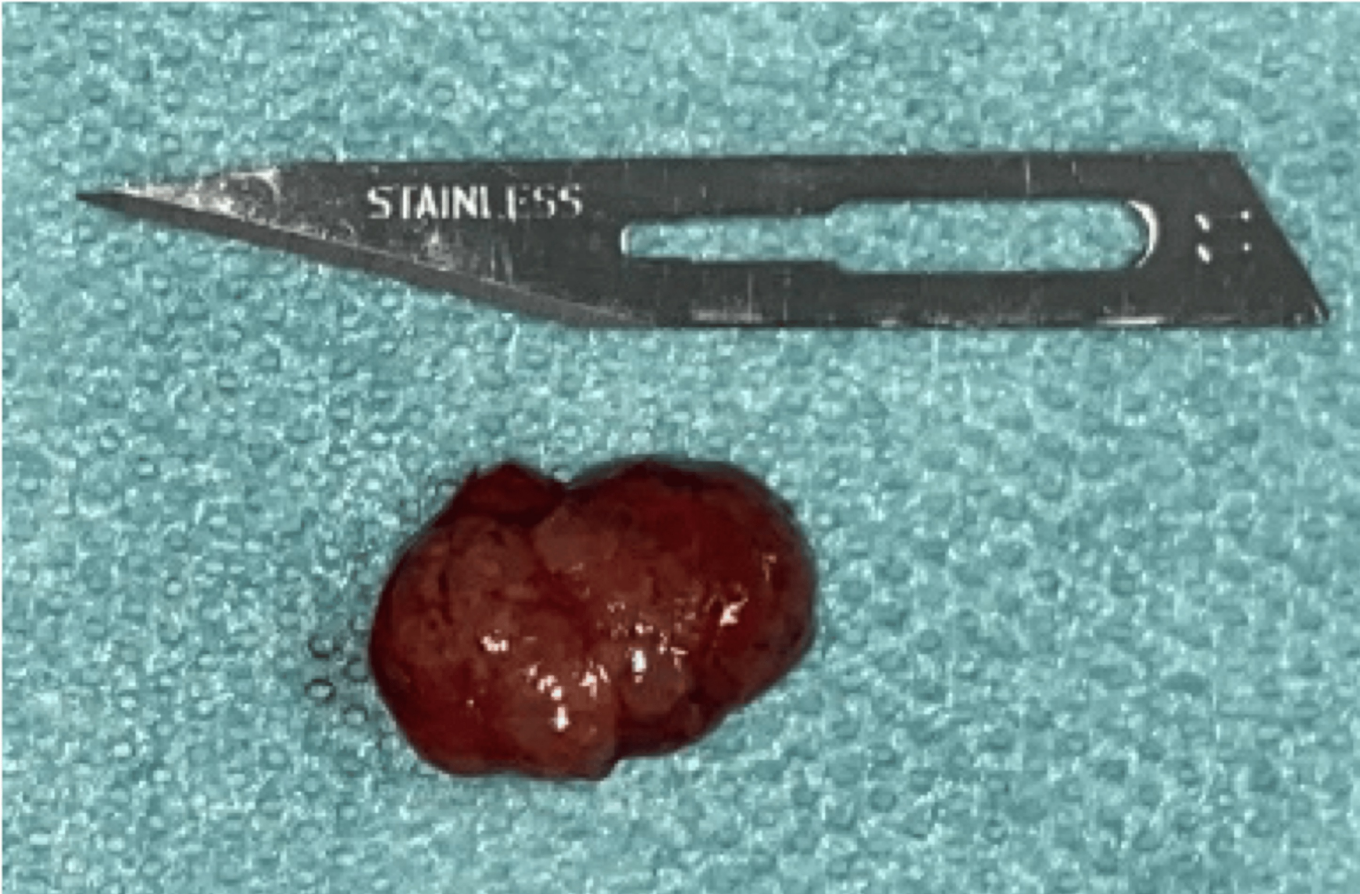
Anatomical specimen

The patient underwent an excisional biopsy (Figures 8, 9). The anatomopathological exam showed a well-limited proliferation with a thin fibrous capsule, with cubic cells with abundant eosinophilic and rounded cytoplasm of monomorphic appearance. Immunohistochemical analysis demonstrated positive staining for both CK7 and P63 antibodies. The anatomopathological examination confirmed BCA. A follow-up after three years did not show any signs of recurrence.

## Discussion

BCAs are benign tumors that affect the salivary glands and account for 1-3% of all salivary tumors. They are generally found in the major glands, in particular the parotid gland, in 75% of cases, followed by the submandibular gland in 5% of cases. Their location in the accessory glands remains very rare [2]. When the tumor is located in the accessory salivary glands, in 40-80% of cases, it is found in the palate [3]. To better understand the characteristics of these tumors, a literature review spanning the period from 2005 to 2025 was conducted using the Medline database. The search was performed using the keywords “basal cell” AND “adenoma” AND “oral”. Twenty-three relevant articles were identified, leading to the selection of 28 cases of BCA (Table 1).

**Table 1:** Summary of the reported cases of basal cell adenoma of the oral region

Case no.	Study	Age/sex	Location	Tumor size (cm)	Histological pattern	Treatment and recurrence
1	Mărgăritescu et al., 2005 [15]	55/M	Upper lip	2 x 2	Solid	Not determined
2	Mărgăritescu et al., 2005 [15]	57/F	Upper lip	1.5 x 2	Solid	Not determined
3	Minicucci et al., 2008 [16]	51/F	Upper lip	4 x 4	Solid	Excision, no recurrence (6 months)
4	Antoniades et al., 2009 [17]	68/M	Upper lip	1.5 x 1	Solid	Excision, no recurrence (5 years)
5	Antoniades et al., 2009 [17]	67/M	Upper lip	0.5 x 0.5	Solid	Excision, no recurrence (10 months)
6	Gupta et al., 2009 [18]	32/M	Palate	3.5 x 2	Solid/trabecular/membranous	Excision, no recurrence
7	Soares et al., 2010 [19]	69/M	Upper lip	1.5 x 1	Trabecular/tubular	Surgical excision, no recurrence after 2 years
8	Ishibashi et al., 2012 [20]	68/M	Palate	2.2 x 3.2	Solid/trabecular/tubular	Excision, no recurrence (3.5 years)
9	Kudoh et al., 2014 [21]	59/M	Upper lip	1 x 1	Trabecular/tubular	Excision, no recurrence
10	Gupta et al., 2014 [22]	42/M	Upper lip	1 x 1	Trabecular/tubular	Excision
11	Bhagat et al., 2015 [23]	52/M	Palate	ND	Solid	Excision, only 1 recurrence (membranous type)
12	Bhagat et al., 2015 [23]	28/F	Upper lip	ND	Membranous	Not determined
13	Bhagat et al., 2015 [23]	42/F	Upper lip	ND	Tubular	Not determined
14	Bhagat et al., 2015 [23]	52/M	Buccal mucosa	ND	Solid	Not determined
16	Beanes et al., 2015 [24]	76/F	Upper lip	1.1 x 1	Trabecular/tubular	Surgical excision, no recurrence after 8 years​
17	Karim, 2015 [25]	84/M	Upper lip	2 x 1	Trabecular/tubular	Excision
18	Sodhi et al., 2015 [26]	25/M	Palate	3 x 4	Solid/tubular	Excision, no recurrence
19	Yadav et al., 2015 [7]	55/F	Palate	2.5 x 2	Solid/tubular	Excision, no recurrence after 1 year
20	Krishnan et al., 2016 [27]	45/F	Retromolar region	2 x 2	Trabecular/tubular	Excision, no recurrence
21	Jeddy, 2017 [28]	61/F	Buccal mucosa	3 x 2	Membranous	Excision planned, patient lost to follow-up
22	Ramesh et al., 2017 [29]	45/M	Palate	ND	Trabecular	Not determined
23	Tatehara et al., 2019 [30]	73/F	Upper lip	2.2 x 1	Trabecular/tubular	Excision, no recurrence (16 years)
24	Sirohi et al., 2020 [31]	64/F	Sublingual gland	3.0 × 2.0	Trabecular/tubular	Excision, no recurrence
25	Takeuchi et al.,2022 [32]	41/M	Alveolar region anterior mandible	1.2 x 1.0	Solid/tubular	Excision, no recurrence (1.5 years)
26	Taketomiet al., 2022 [33]	47/F	Buccal mucosa	1.5 x 1.5	Solid/trabecular/tubular	Excision, no recurrence (3 years)
28	Alrohaimi et al., 2023 [5]	45/F	Buccal mucosa	1.9 x 1.5	Solid	Excision, no recurrence
29	Yoshimura et al., 2024 [34]	70/F	Palate	3.2 × 2.6	Trabecular/tubular	Excision, no recurrence after 3.5 years
30	Ahmad et al., 2025 [35]	48/F	Upper lip	1.2 x 1	Solid	Excision, no recurrence after 1 year
31	Present case	58/M	Upper lip	2 x 3	Trabecular/tubular	Excision, no recurrence
32	Present case	75/F	Upper lip	1 x 3	Trabecular/tubular	Excision, no recurrence

BCA was classified in 1972 by the WHO among monomorphic tumors distinguished by its uniform cellular morphology in contrast to pleomorphic adenoma, which presents various histologic features. In 1991, it was described as a benign epithelial neoplasm entity with four subtypes. This classification was retained in both the 2017 and 2022 WHO classifications, as the latter did not report any changes regarding the composition of BCAs [4,5,6]. BCAs are categorized into four histopathological types: solid, tubular, trabecular, and membranous, depending on their predominant architectural features. The solid form is the most frequently encountered variant. Our reported cases demonstrated the trabecular variant, one of the recognized histological patterns. In all cases, surgical excision was the treatment of choice, with no recurrences noted, reinforcing the benign nature and favorable prognosis of BCA. It mainly affects people with an average age of 50 years or more. Women are affected more than men [5,6,7].

The literature review showed a slight male predominance with a male-to-female ratio of approximately 1.5:1. This observation aligns with our series, which included one male and one female case. The age of affected patients ranged from 25 to 84 years, with a predominance in middle-aged and older adults, again consistent with our cases aged 58 and 75 years. Clinically, BCA presents in the form of a well-defined nodule with slow growth, painless, and covered with a normal-appearing mucosa, as illustrated in our two cases [6,7,8]. The upper lip emerged as the most frequent site of involvement in minor salivary glands, followed by the palate and buccal mucosa. These findings are consistent with the predilection of BCA for minor glands. Tumors typically presented as small, well-circumscribed nodules ranging in size from 1 to 3 cm.

Histologically, BCA is characterized by the presence of two cell types. The first cell type is small, cuboidal or columnar shaped, present peripherally in a palisading arrangement within the tumor nests or cords, with round, deeply stained nuclei and little discernible cytoplasm. The second cell type, presenting centrally, is larger with modest cytoplasm, indistinct cell borders, and a pale-staining oval nucleus. Sharp demarcation is present between the neoplastic cells and the surrounding stroma [7]. The differentiation between BCA and BCAC is thus based on the demonstration of peripheral invasion, vascular emboli, perineural sheathing, and a greater number of mitoses. The presence of cellular pleomorphism, mitotic figures, high nuclear cytoplasmic ratio, prominent large nucleoli, and necrosis on cytological smears strongly indicates malignancy [1,2,9,10].

According to the 2022 classification of salivary gland tumours, most BCACs develop de novo [11]. The differential diagnosis with pleomorphic adenoma is made based on the absence of myxoid and/or chondroid stroma [12]. Canalicular adenoma is a tumor of the salivary glands that can also be confused with the BCA. Rarely found in patients under 50 years, it is histologically characterized by a double row of cylindrical cells, which can be tight or separated, forming canaliculi within a loose stroma [13]. However, immunohistochemical marking is the principal way to differentiate between BCA and canalicular adenoma, mainly by highlighting P63, which would be negative in canalicular adenomas and positive in BCAs, P63 being a selective marker of myoepithelial cells and basal cells. Immunohistochemical marking, on the other hand, does not confirm the malignancy of the lesion [2,14].

Both of our cases underwent an immunohistochemical analysis with P63. In both cases, surgical excision was the treatment of choice, with no recurrences noted, reinforcing the benign nature and favorable prognosis of BCA. However, a recurrence after an incomplete excision is possible. A rate of recurrence of 25-37% for the membranous subtype has been reported. Malignant degeneration is rare [5,6,7].

## Conclusions

While most tumors of the accessory salivary glands are pleomorphic adenomas, the diagnosis of a BCA should not be ruled out. Although BCA is a rare entity, it is important to take it into account in the differential diagnosis, and clinicians should perform a careful histopathological examination to rule out BCAC. BCAC has histological characteristics that overlap with BCA. BCA recurrences are rare, but proper follow-up must be performed in these patients.
